# Systematic Analysis and Identification of Dysregulated Panel lncRNAs Contributing to Poor Prognosis in Head-Neck Cancer

**DOI:** 10.3389/fonc.2021.731752

**Published:** 2021-10-18

**Authors:** Shang-Ju Tang, Guo-Rong You, Joseph T. Chang, Ann-Joy Cheng

**Affiliations:** ^1^ Department of Medical Biotechnology and Laboratory Science, College of Medicine, Chang Gung University, Taoyuan, Taiwan; ^2^ Graduate Institute of Biomedical Sciences, College of Medicine, Change Gung University, Taoyuan, Taiwan; ^3^ Department of Radiation Oncology, Chang Gung Memorial Hospital, Taoyuan, Taiwan; ^4^ Department of Medical School, College of Medicine, Chang Gung University, Taoyuan, Taiwan

**Keywords:** lncRNA - long noncoding RNA, head and neck cancer, prognostic panel, XIST (X-inactive specific transcript), altered gene expression

## Abstract

Head and neck cancer (HNC) is one of the most prevalent cancers worldwide, accounting for approximately 5% of all cancers. While the underlying molecules and their pathogenetic mechanisms in HNC have yet to be well elucidated, recent studies have shown that dysregulation of lncRNAs may disrupt the homeostasis of various biological pathways. However, the understanding of lncRNAs in HNC is still limited by the lack of expression profiling. In the present study, we employed a systematic strategy to identify a panel of lncRNA associated with HNC. A cancer-related lncRNA profile PCR array was screened to explore potential molecules specific for HNC. A total of 55 lncRNAs were found to be dysregulated in HNC cells when compared to normal keratinocytes. Further analysis of the prognostic significance using The Cancer Genome Atlas (TCGA) database revealed 15 lncRNAs highly correlated with overall survival in HNC patients. Additionally, clinical sample expression analysis of the TCGA-HNSC cohort revealed 16 highly dysregulated lncRNAs in HNC, resulting in a combined 31-lncRNA signature panel that could predict prognosis. Validation of these molecules confirmed the considerable level of altered expressions in HNC cells, with XIST, HOXA11-AS, TSIX, MALAT1, WT1-AS, and IPW being the most prominently dysregulated. We further selected a molecule from our panel (XIST) to confirm the validity of these lncRNAs in the regulation of cancer aggressiveness. Gene ontology (GO) and KEGG (Kyoto Encyclopedia of Genes and Genomes) pathway enrichment analyses demonstrated that XIST participated in various cancer-related functions, including cell proliferation and metastasis. XIST silencing with the RNAi technique substantially reduced invasion and migration in several HNC cell lines. Thus, our study defined a 31-lncRNA panel as prognostic signatures in HNC. These perspective results provide a knowledge foundation for further application of these molecules in precision medicine.

## Introduction

Head and neck cancer (HNC) is a complex and difficult to treat disease. While this type of cancer encompasses dysregulations at areas including the mouth, nasal cavity, larynx, and pharynx, over 90% of all HNCs are squamous cell carcinomas, and of the oral region (1). Together, they account for approximately 5% of all cancers worldwide, according to GLOBOCAN 2020 estimates (2). Like other cancers, standard treatment methods include surgery, radiotherapy, chemotherapy, or combination therapy (3). Nevertheless, even after months of treatment, relapse is always a potential problem. Although the 5-year survival rate of HNC patients is roughly 80% when detected at the earliest stages, mid-to late-stage detection causes that number to decrease by over two-fold (4, 5). Therefore, it is critical to identify and understand how these carcinogenic mechanisms and molecules affect cancer, as we may be able to uncover the mysteries behind HNC and how to treat and/or prevent it.

It is well established that while more than 75% of the human genome is transcribed, only 2% consists of coding genes (6). The remaining non-coding RNAs, previously merely labeled as transcriptional noise or garbage sequences and disregarded, have recently become much more recognized. The largest class of the non-coding RNA family, with transcripts longer than 200 nucleotides, are known as long non-coding RNAs (LncRNAs). They have gained significant attention over the past decade, as many studies have confirmed their roles in various biological processes involving transcriptional and epigenetic regulation, metabolism, and multiple cellular functions (7–9).

So far, there has been no distinctive markers for HNC. Nevertheless, many studies have shown that aberrantly expressed lncRNAs may potentially play important roles in this particular cancer type. Currently, only a handful of lncRNAs have been implicated in different cancerous functions such as migration, invasion, and metastasis of HNC, including HOTAIR, UCA1, and MALAT1 (10). A variety of lncRNAs have been discovered to play roles in various cancers. For example, lncRNA HOTAIR and UCA1 have both been found to play carcinogenic roles in multiple cancer types (11, 12). A recent review by Zhou et al. also depicted various lncRNAs that were implicated in HNC metastasis (13). Moreover, since not many studies have profiled lncRNAs in cancers of the head and neck region in combination with prognosis analysis, the results of this research will allow us to better understand the mechanisms behind HNC, and provide new insights on the development of diagnostic, prognostic, or treatment markers.

However, the screening and selection of these molecules are mostly ambiguous, and their prognosis abilities have yet to be thoroughly investigated.

The Cancer Genome Atlas (TCGA) is a comprehensive database for identifying and annotating different genes across multiple cancers. With the recent development in genomic sequencing, many cancer-associated lncRNA studies have been accomplished by solely analyzing and constructing data based on these clinical datasets (14–16). While various studies have profiled lncRNAs across different cancers, including breast cancer (17), lung cancer (18), and esophageal cancer (19), very few focus on the intricacies of HNC. Additionally, although the high-throughput TCGA datasets offer a large library of potential candidate molecules, exclusively relying on database information without experimental validation may limit insight for substantiation of prognostic cancer markers, as many have noted (20–24). Therefore, examination of validated lncRNAs in combination with big-data analysis would be ideal.

PCR array is a relatively new method of gene expression analysis. While it may lack discovery power or high-throughput abilities, it makes up for in its sensitivity, specificity, and great dynamic range. Additionally, data analysis is quick and efficient, as opposed to the cumbersome bioinformatics analysis required for genome-wide methods. Previous studies have reported the use of PCR arrays to analyze genes in specific pathways or diseases. For example, Boone et al. performed two pathway-specific arrays for apoptosis and neurotrophins & receptor genes to elucidate the changes in post-traumatic brain injury (25). Zhang et al. also used a panel of 54 genes specific to Alzheimer’s disease to observe changes in gene expression in mice (26).

To our knowledge, there are very few reports that show a systematic profiling investigation of HNC lncRNAs, nor their correlation with prognosis. Herein, we systematically examined differentially expressed lncRNAs in HNC cells using a PCR array-based method. We further assessed our results with prognostic information obtained from a high-throughput database, the TCGA-HNSC cohort. Additionally, expression levels of the top dysregulated lncRNAs from the same TCGA dataset were parallelly assessed to provide a base foundation for our research. In combination with *in silico* and *in vitro* analysis, we defined a panel of 31-lncRNA signatures with valuable prognostic information.

## Materials and Methods

### Cells and Cell Cultures

A total of 10 HNC cell lines, SAS, OECM1, FaDu, Detroit, SCC4, SCC25, OC3, BM1, BM2, and NPC076, and six normal keratinocyte cell lines, CGHNK2, CGHNK4, CGHNK6, CGHNK16, CGHNK47, and NOK were used. Cells are cultured and maintained as previously described (27). Briefly, SAS and NPC076 cells were maintained in Dulbecco’s Modified Eagle Medium (DMEM, Gibco^®^), SCC4 and SCC25 cells were maintained in DMEM/F12 medium (D-MEM/F-12, Gibco^®^), OECM1, BM1, BM2, and KYSE cells were maintained in Roswell Park Memorial Institute 1640 medium (RPMI 1640, Gibco^®^), FaDu and Detroit cells were maintained in Minimum Essential Media (MEM, Gibco^®^), OC3 was maintained in 1:2 DMEM/Keratinocyte Serum-Free Medium (KSFM, Gibco^®^), and normal keratinocyte cell lines were cultured in KSFM (KSFM, Gibco^®^). Cancer cell line mediums were supplemented with 7% FBS and 1% Antibiotic-Antimycotic, and all cells were incubated at 37°C in a humidified atmosphere of 5% CO_2_.

### LncRNA Screening *via* RT^2^ PCR Array

Systematic gene profiling was accomplished using Qiagen’s PCR array kit, according to the manufacturer’s protocol. Briefly, RNA from cell pellets were extracted and quantified. cDNA synthesis was performed using the RT^2^ First Strand Kit (Cat. No. 330401; Qiagen, GmbH), and subsequently combined with the RT^2^ SYBR^®^ Green PCR master mix (Cat. No. 330504; Qiagen, GmbH). The master mix was then used in combination with the Human Cancer PathwayFinder RT^2^ lncRNA PCR Array (Cat. No. LAHS-002Z; Qiagen, GmbH), and the output data was analyzed using the GeneGlobe Data Analysis Center at http://www.qiagen.com/geneglobe.

### LncRNA Analysis *via* RT-qPCR

Cell pellets were washed with PBS and collected for RNA isolation. Total RNA extraction was performed using TRIzol reagent (Gibco BRL), and quantification was achieved with a Nanovue™ spectrophotometer (GE Healthcare). cDNA synthesis was achieved by combining total RNA (2 μg) with 5x first-strand buffer (GeneDireX, Inc.), 0.1M DTT (Invitrogen; Thermo Fisher Scientific, Inc.), 1 unit of RNase inhibitor (Invitrogen; Thermo Fisher Scientific, Inc.), 25 mM dNTPs (Thermo Scientific, Thermo Fisher Scientific, Inc.), and random hexamer primers to a total reaction volume of 30μl. TaqMan qPCR assay kit (Applied Biosystems, Thermo Fisher Scientific, Inc.) was combined with the cDNA to create a 20 μL reaction volume to measure lncRNA expression after 50 cycles. For SYBR green reactions, iQ™ SYBR^®^ Green Supermix (Bio-Rad, Inc.) was used instead. Specific lncRNA PCR primers were designed through primer blast. The PCR primers used in this study are listed in **Supplementary Table S1**. Results were normalized against GAPDH internal control.

### Western Blot Analysis of lncRNA Targets

Cell lysates were isolated by homogenization in CHAPS lysis buffer (10 mM Tris, pH 7.4, 1 mM MgCl2, 1 mM EGTA, 150 mM NaCl, 0.5% CHAPS and 10% glycerol; Sigma-Aldrich; Merck KGaA) containing a protease and phosphatase inhibitor. Briefly, cell pellets were resuspended in ice-cold buffer and incubated on ice for 30 minutes. Protein collection *via* centrifugation at 13000 g for 30 minutes at 4°C was performed, followed by protein concentration quantification with Bradford assay (Bio-Rad, Inc.), according to the manufacturer’s instructions. Protein separation was performed using 10% sodium dodecyl sulfate-polyacrylamide (SDS-PAGE) gel with 30 μg of protein and transferred onto a nitrocellulose membrane. 5% milk was used for blocking, and specific primary antibodies were hybridized overnight at 4°C. Subsequently, the membranes were incubated with secondary antibodies and visualized through chemiluminescent detection. GAPDH was used as an internal control.

### Clinical Evaluation of LncRNAs Related to Prognosis in HNC Patients

The RNA-seq data was obtained through the UALCAN web portal (http://ualcan.path.uab.edu/) (28). The Kaplan-Meier survival curves were plotted to evaluate the prognosis of lncRNAs in high- and low-risk patient groups. The head and neck RNA-seq dataset on the Kaplan-Meier Plotter pan-cancer database (https://kmplot.com/analysis/) was selected to assess the overall survival of lncRNAs. Head-neck squamous cell carcinoma data was collected from sources including TCGA, European Genome-phenome Archive (EGA), and Gene Expression Omnibus (GEO) (n = 500), and cohorts were split by automatic sel7best cut-off for median expression values. Additionally, univariate proportional cox hazard ratios (HRs) with 95% confidence intervals, along with survival p-values calculated by log-rank test were obtained for each lncRNA. TCGA clinical expression level analysis was performed using SurvExpress (http://bioinformatica.mty.itesm.mx:8080/Biomatec/SurvivaX.jsp), and pan-cancer analysis was performed with Gepia2 (http://gepia2.cancer-pku.cn/#index).

### Molecular Targets and Pathway Analyses *via* Bioinformatic Methods

Potential lncRNA-binding axes were investigated through various online databases and prediction algorithms. Potential lncRNA-mRNA or protein bindings were screened using ENCORI (version 3.0, http://starbase.sysu.edu.cn/index.php) (29). Gene Ontology (GO) and pathway analysis were conducted with the GO and KEGG database from The Database for Annotation, Visualization and Integrated Discovery (DAVID, version 6.8, https://david.ncifcrf.gov/).

### Knockdown LncRNA XIST Expression *via* Specific siRNA Transfection

Knockdown of lncRNAs was accomplished with specific siRNAs (Thermo Scientific, Thermo Fisher Scientific, Inc.). siRNA sequences are listed in **Supplementary Table S1**. Transfection was performed with Lipofectamine 2000™ reagent (Invitrogen, Thermo Fisher Scientific, Inc.) in OPTI-MEM medium (Invitrogen, Thermo Fisher Scientific, Inc.), according to the manufacturer’s instructions. Briefly, cells were seeded in a 10-cm dish and incubated for 24 hours prior to transfection. 20 to 40 µg of siRNA was transfected and incubated for another 24 hours before the cells were counted and/or pelleted for functional assay examination.

### Determination of Cellular Functions: Growth, Migration, and Invasion

Colony formation assay was performed by seeding 5 x 10^2^ to 5 x 10^3^ transfected cells into 6-well plates and incubated without disturbance for 10 to 14 days. The cells were then fixed and stained with crystal violet for 2 hours, and the number of colonies formed was counted.

Cell invasion was performed using Millicell^®^ (Millipore) cell culture inserts. Transwell chambers were coated with matrigel, and 1 x 10^6^ transfected cells were seeded into the upper chamber. The lower chamber was filled with 20% FBS culture medium to promote cell invasion. After 16 to 24 hours of incubation at 37°C, invaded cells were fixed with formaldehyde for 30 minutes, stained with crystal violet for 2 hours, and the number of invaded cells which passed through the Matrigel-coated membranes were quantified and compared to their control counterparts.

Cell migration assay was performed using the wound-healing method. 1 x 10^5^ transfected cells were seeded into Ibidi^®^ culture inserts for 16 hours. After the cells adhered, the inserts were removed, leaving a cell-free gap in the monolayer of cells. Migration towards the gaps was then photographed and measured at 4-hour intervals, up to 12 hours.

### Statistical Analysis

RT-qPCR data was performed with at least three independent experiments for each experimental cohort. Unpaired t-test was used to compare the normal and cancer groups (Graphpad Prism 8.0), and a p-value of ≤ 0.05 was considered statistically significant.

## Results

### LncRNA Expression Profiling in HNC Cell Lines

**Figure 1:**
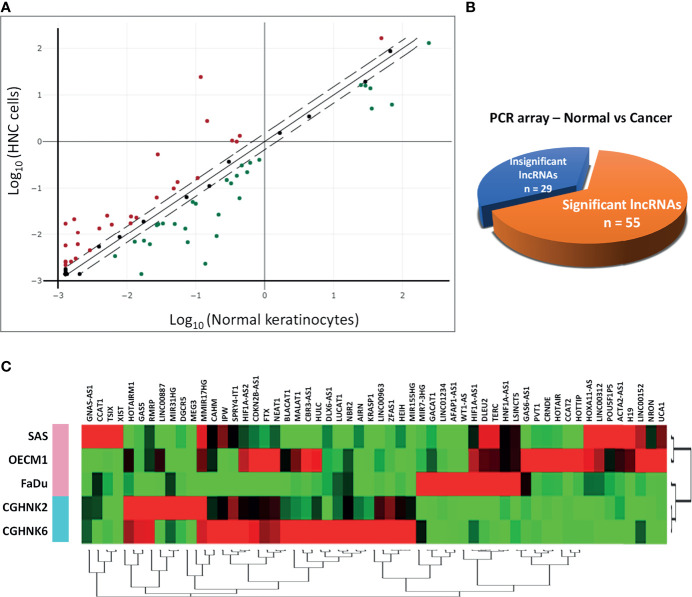
Profiling of 84 lncRNAs in three HNC cell lines (SAS, OECM1, FaDu) versus two normal keratinocyte cell lines (CGHNK2, CGHNK6) *via* lncRNA PCR Array. **(A)** Scatterplot of 84 lncRNAs. Dotted lines represent the selection criteria threshold of |FR| ≥ 1.5. Red dots represent upregulated lncRNAs (n = 27), and green dots represent downregulated lncRNAs (n = 28). Black dots represent insignificant lncRNAs according to the selection criterion (n = 29). **(B)** Pie chart representation of the screening composition between significantly dysregulated lncRNAs (n = 55) and insignificant lncRNAs (n = 29) in HNC, according to the PCR array results. **(C)** Clustergram of the 55 significantly dysregulated lncRNA expression profiles between the HNC and normal keratinocyte cell groups.

To profile lncRNAs associated with HNC, a PCR array with 84 cancer-related lncRNAs was used to examine the differential expressions between three HNC cell lines (SAS, OECM1, and FaDu) and two lines of normal keratinocytes (CGHNK2 and CGHNK6). The three HNC cell line’s geometric mean fold regulation (FR) and fold change (FC) of each lncRNA was compared to the mean of the normal cell lines, as summarized in **Supplementary Table S2**, and the relationship between cancerous and normal groups were shown in **Figure 1A**. A screening criterion of a mean |FR| ≥ 1.5 was established, resulting in 55 significantly dysregulated lncRNAs (**Figure 1B**). Among these, 27 lncRNAs were upregulated, and 28 were downregulated (**Figure 1B** and **Tables 1**, **2**). Hierarchical clustering analysis was used to visualize these differentially expressed lncRNAs (**Figure 1C**). These results suggest a panel candidate of lncRNA that may participate in the carcinogenesis of HNC.

**Table 1:** List of upregulated genes across the HNC cell line group compared with the normal keratinocyte cell line group.

Up-Regulation (n = 27, comparing to control group)					
Symbol	SAS	OECM1	FaDu	Geometric mean
	Fold Regulation	Fold Regulation	Fold Regulation	Fold Regulation
H19	895.97	612.4	16.05	206.51
UCA1	43.33	58.42	2.68	18.94
CRNDE	10.36	65.04	9.98	18.88
XIST	1785.75	-1.03	1.38	13.41
HOTAIR	6.33	43.81	7.01	12.48
NRON	7.4	16.89	1.43	5.63
LINC00312	7.17	6.12	2.77	4.95
HOXA11-AS	6.16	5.83	2.46	4.45
LINC01234	-3.34	3.06	43.4	3.41
GACAT1	1.91	2.66	6.15	3.15
PVT1	2.05	9.6	1.53	3.11
LINC00152	4.48	5.22	1.21	3.05
LSINCT5	2.4	2.42	3.48	2.72
TERC	2.68	2.18	2.81	2.54
DLEU2	2.62	2.16	2.74	2.49
GAS6-AS1	-1.75	6.03	3.63	2.32
DLX6-AS1	2.59	1.91	1.99	2.14
AFAP1-AS1	-1.17	-1.05	10.78	2.06
WT1-AS	-1.08	-1.03	8.87	2
HOTTIP	-1.08	5.63	1.38	1.93
CCAT1	8.77	-6.76	3.71	1.69
TSIX	3.51	-1.03	1.38	1.68
HIF1A-AS1	1.32	1.78	1.94	1.66
HNF1A-AS1	1.5	1.51	2.02	1.66
ACTA2-AS1	2.14	1.66	1.05	1.55
CCAT2	-1.63	10.65	-1.77	1.55
POU5F1P5	2.14	1.47	1.18	1.55

**Table 2:** List of downregulated genes across the HNC cell line group compared with the normal keratinocyte cell line group.

Down-Regulation (n = 28, comparing to control group)					
Symbol	SAS	OECM1	FaDu	Geometric mean
	Fold Regulation	Fold Regulation	Fold Regulation	Fold Regulation
KRASP1	-87.59	-31.38	-77.46	-59.71
IPW	1.07	-101.59	-113.01	-22.08
MEG3	-13.62	-12.97	-9.13	-11.73
MIR155HG	-13.86	-10.28	-10.38	-11.39
ZFAS1	-7.61	-8.15	-23.43	-11.33
SPRY4-IT1	-1.65	-13.56	-22.55	-7.96
LINC00963	-14.45	-3.37	-7.39	-7.11
GAS5	-5.79	-5.47	-10.82	-7
BLACAT1	-12.98	-1.22	-9.07	-5.24
DGCR5	-2.11	-6.01	-3.53	-3.55
LUCAT1	-6.18	-2.63	-1.53	-2.92
MALAT1	-6.72	1.35	-3.41	-2.57
HEIH	-1.92	-3.32	-2.58	-2.54
LINC00887	-6.2	2.11	-4.98	-2.45
HIF1A-AS2	-1.78	1.12	-6.39	-2.16
MIR17HG	-1.02	-1.28	-7.6	-2.15
MIR31HG	-2.63	-2.61	-1.28	-2.07
GNAS-AS1	2.55	-5.38	-3.79	-2
CDKN2B-AS1	-1.93	1.33	-4.89	-1.92
HOTAIRM1	-2.71	-1.29	-1.93	-1.89
HULC	-3.67	1.36	-2.5	-1.89
RMRP	-1.63	-1.58	-2.58	-1.88
CAHM	-1.2	-1.36	-3.51	-1.79
NBR2	-2.63	-1.49	-1.4	-1.76
CBR3-AS1	-3.17	1.68	-2.63	-1.71
AIRN	-1.38	-1.89	-1.89	-1.7
FTX	-1.76	1.29	-2.59	-1.52
NEAT1	-1.56	1.26	-2.84	-1.52

### Prognostic Significance of the Panel lncRNAs in HNC Patients

**Figure 2:**
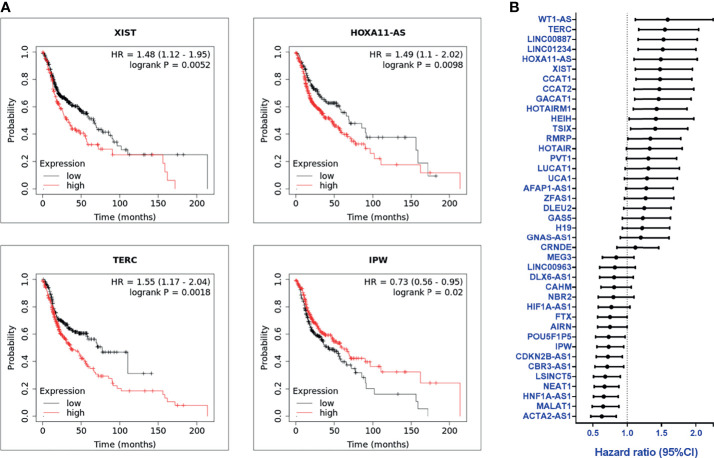
Overall survival and prognosis analysis of profiled lncRNAs **(A)** Kaplan-Meier survival curve examples of dysregulated lncRNAs in HNC. Hazard ratios (HRs) were calculated with 95% confidence intervals (CI), and p-values ≤ 0.05 were considered significant. **(B)** HRs of 41 significantly dysregulated lncRNAs. A total of 41 lncRNAs were examined, post-exclusion of nine lncRNAs from the 55 highly dysregulated candidates with no information provided in KM plotter.

The clinical significance of the lncRNA in HNC patients was determined by examining the association between each lncRNA expression and clinical patients’ prognosis. The Kaplan-Meier Plotter (KM Plotter) suite was used to analyze overall survival in HNC patients with the TCGA-HNSC dataset (n=500). A total of 41 lncRNAs was examined, which included the 55 candidates post-exclusion of nine molecules without information in KM Plotter. **Figure 2A** shows a few examples of highly significant results. As depicted, the upregulated lncRNAs, XIST, HOXA11-AS, and TERC, were significantly associated with poor prognosis, while IPW, a downregulated lncRNA, was correlated with good survival. The hazard ratio (HR) of the prognostic association for each lncRNA was summarized in **Figure 2B**. In total, there were 24 lncRNAs with HR ≥ 1.0, implying the higher risk of these lncRNA expressions to be correlated with worse survival, whereas 17 lncRNAs were found with HR < 1.0, alluding to a lower lncRNA level, favoring good prognosis in HNC patients.

### Dysregulated lncRNA Signatures in Cells Correlated With Prognosis in Patients

**Figure 3:**
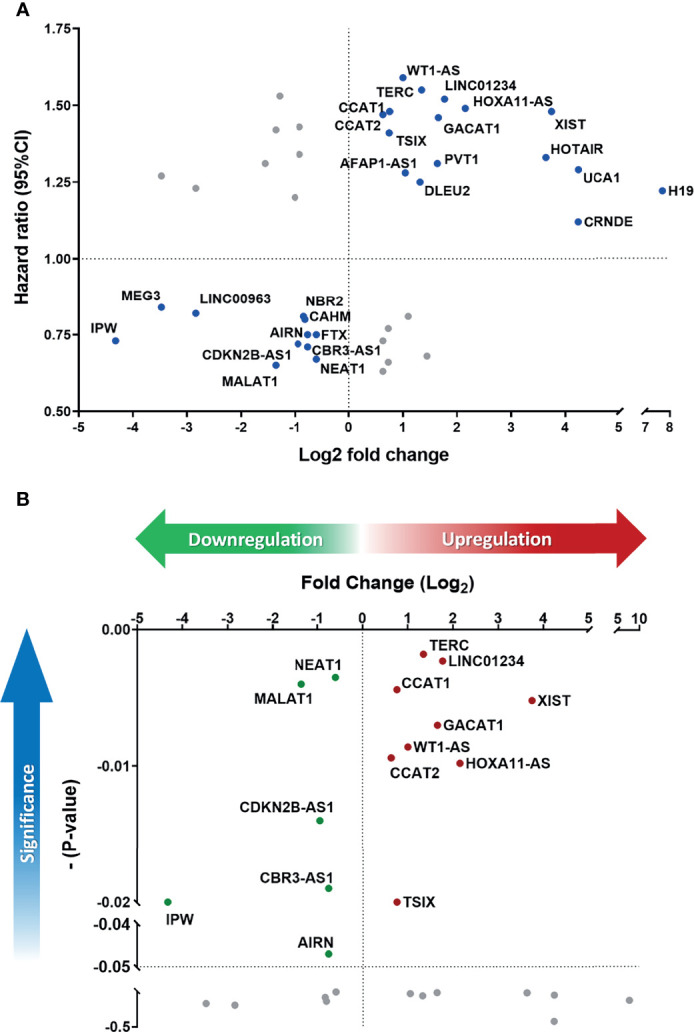
Defining the lncRNA panel based on correlation between gene expression and prognostic information. **(A)** HR versus log_2_-fold change (FC) dot-plot of 41 significantly dysregulated lncRNAs from the PCR array. In total, 27 lncRNAs were correlated with high risk score. **(B)** Overall survival p-value versus log_2_-FC of 27 positively correlated lncRNAs. A total of 15 lncRNAs were significantly correlated with cancer prognosis (p-value ≤ 0.05).

To parallelly assess lncRNA expression level and the prognostic significance in HNC patients, **Figure 3A** was plotted to show the association between these two parameters of each molecule. As shown, a total of 27 lncRNAs exhibited correlative levels of dysregulation and prognostic risk (HRs), with 16 being positive-risk and 11 negative-risk to the prognosis of HNC. To further assess the prognostic prediction power, the statistical significance on the overall survival of these 27 lncRNAs was examined. **Figure 3B** depicts the 15 molecules that exhibited altered expression and statistical correlation (p-value ≤ 0.05) in HNC patients. Of these lncRNAs, 9 molecules were upregulated and associated with poor prognosis, including TERC, LINC01234, CCAT1, XIST, GACAT1, WT1-AS, CCAT2, HOXA11-AS, and TSIX. In contrast, a total of 6 molecules, NEAT1, MALAT1, CDKN2B-AS1, CBR3-AS1, IPW, and AIRN, were downregulated and related to good prognosis.

### Prognostic Significance of the Top 30 Up- and Down-Regulated lncRNAs From TCGA-HNSC Database

**Figure 4:**
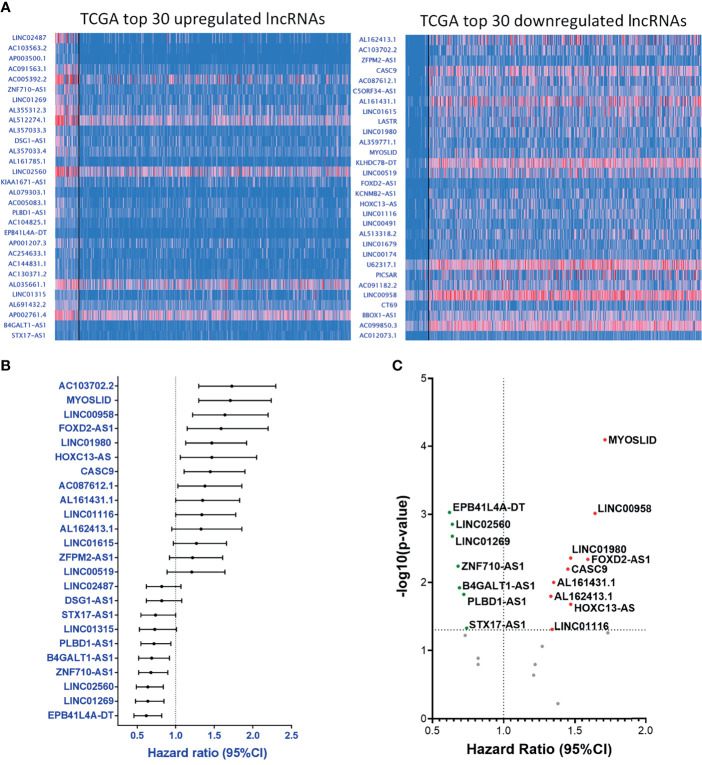
Parallel prognostic significance analysis of clinical samples based on the top 60 dysregulated lncRNA expressions from TCGA-HNSC dataset by UALCAN. **(A)** RNA-seq data of the top 30 upregulated (left) and downregulated (right) lncRNAs were analyzed and depicted through the UALCAN resource. **(B)** HRs of 24 significantly dysregulated lncRNAs. A total of 34 lncRNAs were examined, post-exclusion of lncRNAs with incomplete prognostic information. **(C)** -(Log_10_-p-value) versus HR volcano plot of the 24 significantly dysregulated lncRNAs. A total of 16 lncRNAs were significantly correlated with cancer prognosis (p-value ≤ 0.05).

Apart from the data collected from our PCR array panel, we also analyzed the clinical RNA-seq data from TCGA. Here, we selected the 30 most upregulated and the 30 most downregulated lncRNAs in the TCGA-HNSC cohort (**Figure 4A**). Out of the top 60 dysregulated lncRNAs, we found 24 lncRNAs with HRs significantly correlating with their expression levels, including 14 upregulated lncRNAs with corresponding HRs ≥ 1.0, and 10 downregulated lncRNAs displaying HRs < 1.0 (**Figure 4B**). Further investigation of these molecules revealed 16 genes with p-values ≤ 0.05, signifying a high correlation with prognosis (**Figure 4C**). The collective correlated HRs and significant p-values of the lncRNAs screened are summarized in **Supplementary Table S3**. In combination with our panel derived from the PCR array results, we established a comprehensive panel of 31-lncRNA signatures associated with HNC prognosis.

### A Panel of 31-lncRNA Signature Can Potentially Predict HNC Prognosis

**Figure 5:**
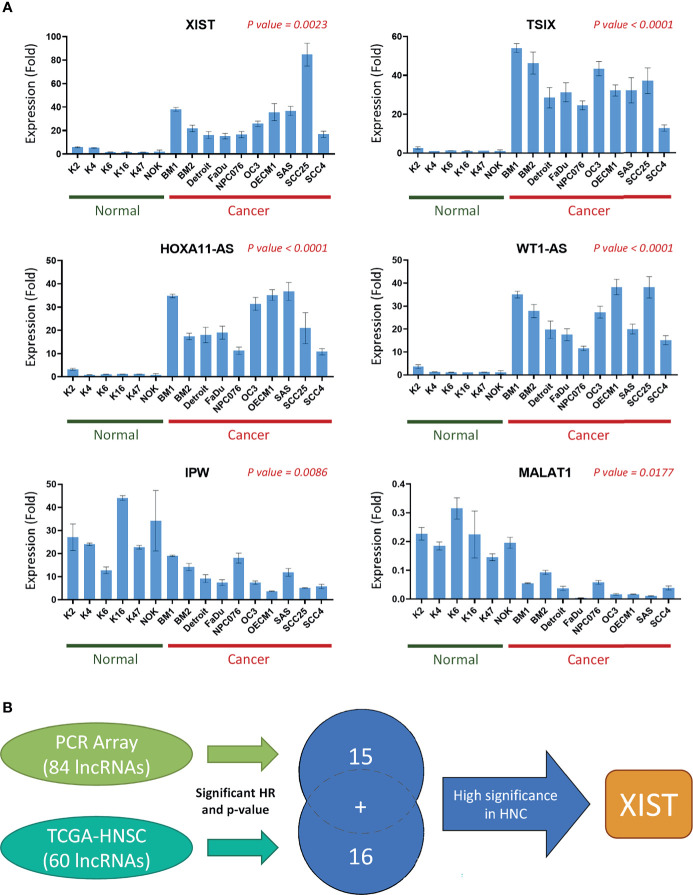
A panel of 31-lncRNA signature predicts the prognosis of HNC. **(A)** The 15 PCR array Panel lncRNAs were verified with various HNC cell lines (n = 10) and normal keratinocytes (n = 6) using RT-qPCR. The six most significantly dysregulated lncRNAs are shown. P-values were calculated using t-test, where p-value ≤ 0.05 was considered significant. **(B)** Schematic flowchart of the systematic screening process. A total of 31 lncRNAs were found to be significantly correlated with HNC prognosis, including 15 lncRNAs found through the PCR array, and 16 found through TCGA database analysis.

Since the expression levels of the lncRNAs from the TCGA dataset are already established from clinical HNC patients, we wanted to specifically confirm and authenticate the extensive capabilities of the 15 lncRNA signatures screened from our own PCR array sample set through RT-qPCR expression analysis. In addition to the five HNC and normal cell lines used in the PCR array, we also included four additional normal cell lines and seven additional cancer cell line samples. **Figure 5A** shows the expression folds of six panel lncRNAs: HOXA11-AS, IPW, MALAT1, TSIX, WT1-AS, and XIST, with statistically significant dysregulation in HNC cells compared to normal cells. As seen in the example lncRNAs shown, the panel lncRNAs are verified across multiple HNC cell lines to be significantly correlated with our PCR array results. Thus, our defined 31-lncRNA signature panel provides insight on dysregulation in cells, as well as correlation with prognosis in HNC patients.

### LncRNA XIST Is Significantly Correlated With HNC

Herein, we selected lncRNA XIST for further functional analysis, due to its significant FR and correlation with TCGA datasets (**Figure 3B** **)**, as well as its high endogenous expression level in HNC cell lines. **Figure 5B** is a schematic representation of our screening and selection. To further verify the potential significance of XIST in cancer, we confirmed its expression in clinical sample data from TCGA (**Supplementary Figure S1A** **)**. Furthermore, we examined its pan-cancer expression. TCGA clinical samples and tissues from various other carcinomas, such as lung, liver/bile duct, and thyroid cancers showed that XIST was upregulated in multiple cancers (**Supplementary Figure S1B**). Additionally, the pan-cancer overall survival for XIST was seen to correlate with poor prognosis, with an HR of 1.2 and a p-value of 0.023, signifying high expression risk, resulting in poor prognosis (**Supplementary Figure S1C**). The data collected from clinical resources coincided with our results with XIST in HNC, verifying that XIST, as well as our other panel lncRNAs could be significant for prognostic analysis of HNC.

**Figure 6:**
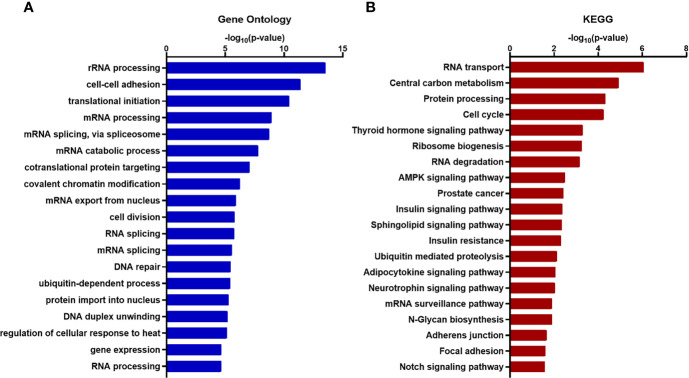
Functional enrichment and annotation of genes associated with XIST *via* bioinformation software. **(A)** GO annotation and **(B)** KEGG pathway enrichment analysis of XIST genes shown in blue and red, respectively. The original significance values obtained from DAVID were transformed to “-log (p-value)” for plotting. Functions and/or pathways with the highest significance are shown.

To acquire comprehensive information related to XIST modulated functional pathway, we performed Gene Ontology (GO) and KEGG (Kyoto Encyclopedia of Genes and Genomes) enrichment analysis and found that many of the genes associated with XIST participate in pathways and functions related to cancer metastasis, including various adhesion-specific functions (**Figures 6A, B** **)**.

**Figure 7:**
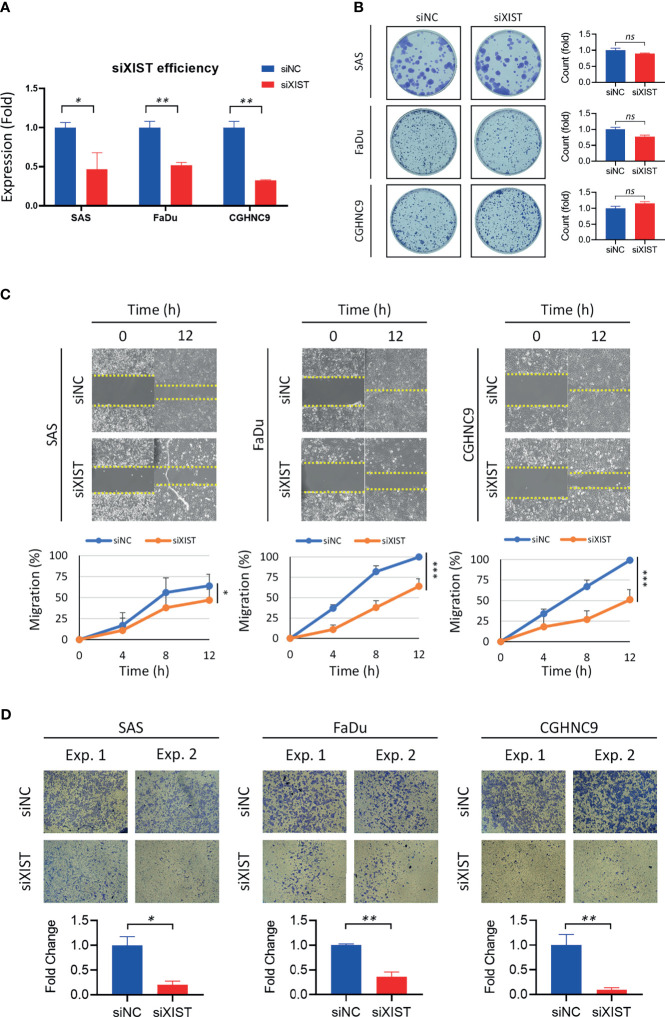
Validation and functional analysis of XIST as an HNC panel biomarker. **(A)** siRNA efficiency of XIST silencing was examined with RT-qPCR. Optimal knockdown was achieved by transfection in SAS, FaDu, and CGHNC9 cell lines. **(B)** Colony formation ability was determined after successful silencing of XIST. No significant difference was observed between the three cancer cell lines when compared to the normal keratinocytes. **(C)** The wound-healing model was used for migration assay. SAS was partially inhibited by roughly 20%, while FaDu and CGHNC9 was inhibited by at least 60%. **(D)** Invasion ability was determined *via* Matrigel invasion assay. All three cell lines showed statistically significant inhibition rates in the XIST knockdown group. All functional experiments were performed in triplicates. (***p ≤ 0.001, **p ≤ 0.01, *p ≤ 0.05, t-test, ns = not significant).

Silencing of XIST was performed with siRNA as the cancer function model (**Figure 7A**). While analysis of long-term cell growth *via* colony formation assay did not show any significant increase or decrease of colony formation ability across different cell lines (**Figure 7B**), both migration and invasion abilities were prominently inhibited when XIST was silenced (**Figures 7C, D** **)**. Migration was partially inhibited in SAS cell lines, while CGHNC9 and FaDu cells had at least a 40% inhibition rate. Similarly, all three cell lines exhibited highly repressed invasion rates in the siRNA group.

**Figure 8:**
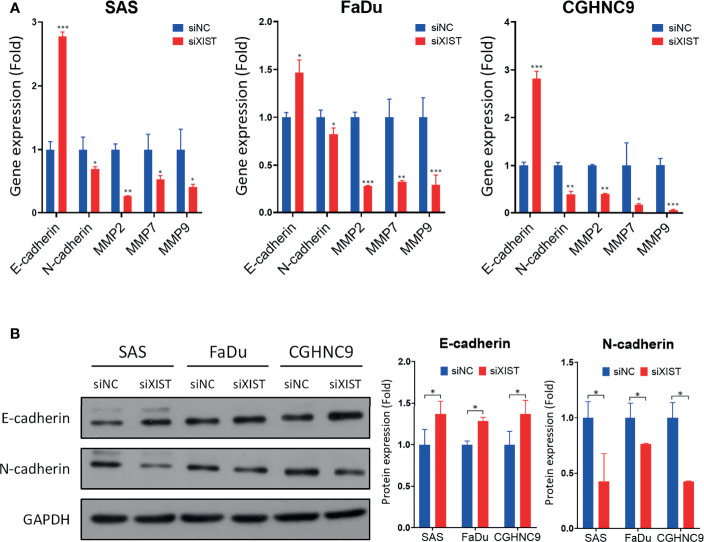
Downstream molecular analysis of XIST-mediated migration and invasion dysregulation. **(A)** RT-qPCR of EMT-associated genes were performed in SAS, FaDu, and CGHNC9 cell lines post-XIST silencing. MMP and mesenchymal markers were downregulated, while the epithelial marker E-cadherin was upregulated. **(B)** Protein expression of EMT markers were analyzed through western blotting. Quantification was achieved using imageJ software. (***p ≤ 0.001, **p ≤ 0.01, *p ≤ 0.05, t-test).

To analyze the downstream mechanisms of XIST-mediated migration and invasion in HNC, we performed RT-qPCR and Western blotting of molecules associated with EMT, including MMP2, MMP7, MMP9, E-cadherin, and N-cadherin. RT-qPCR analysis showed that silencing of XIST also decreased the levels of MMPs and mesenchymal markers, while the epithelial marker E-cadherin was significantly increased across all three cell lines (**Figure 8A**). The protein levels of these genes were also similarly affected. While N-cadherin were inhibited by the knockdown of XIST, E-cadherin was significantly upregulated (**Figure 8B**). Thus, as demonstrated by the analysis of XIST, our panel of lncRNAs can potentially be effectively used as biomarkers that can predict prognosis for HNC.

## Discussion

Cancer has recently become the most common cause of death in higher-income countries. Therefore, it is of utmost importance to find precise molecules that can detect the cancers before it reaches the late stages. LncRNAs are a class of non-coding RNAs that have the ability to modify and/or regulate biological activities, which contributes greatly to all types of diseases, including cancers. A variety of lncRNAs have been implicated from previous studies to play roles in various cancers, including HNC (30). However, to the best of our knowledge, no specific prognostic lncRNA(s) have been derived from a systematically verified study. In this study, we designed a comprehensive strategy to systematically profile prognosis-associated lncRNAs in HNC. A few highlights are noted from our work. [1] Profiling of 84 lncRNAs was performed with a PCR array panel. [2] A total of 55 lncRNAs were found to be highly dysregulated in HNC, with 27 upregulated and 28 downregulated genes. [3] A panel of 31-signature prognosis-associated lncRNAs in HNC was defined. [4] XIST was demonstrated as a critical lncRNA molecule in carcinogenic functions, such as cell migration and invasion. Thus, our defined panel of lncRNAs can be used as potential HNC prognostic markers.

After validating the 84 cancer-associated lncRNAs with PCR array using the criteria |FR| ≥ 1.5, we identified 55 dysregulated lncRNAs in HNC. Many of these lncRNAs have appeared across multiple previous studies. A cancer lncRNA consensus by Carlevaro-Fita et al. listed H19, HOTAIR, MALAT1, and MEG3 as the most prolific lncRNAs, all of which were consistent with our results (31). A review by Cossu et al. also pointed out various lncRNAs, such as AFAP1-AS1, PVT1, MALAT1, H19, DLEU2, CCAT1, and more, that are potentially associated with HNC, many of which also agreed with our findings (32).

Following our initial screening, we investigated the prognostic abilities of these lncRNAs. We chose to evaluate prognosis through HR and overall survival, as the risk of disease in conjunction with time represents imperative determining factors of cancer progression. Univariate cox proportional HRs have been used by multiple studies to represent prognosis potential, as it estimates the relative risk of each lncRNA (33). The results of our HR analysis showed 27 candidates with significant prognosis implications. Then, utilizing overall survival analysis (p-value ≤ 0.05), we evaluated the significance between lncRNA expression and cancer survival. Here, we discovered 15 prognosis-associated lncRNAs. To broaden the scope of our study to include data based on clinical samples, we analyzed highly dysregulated lncRNAs from the TCGA-HNSC dataset in conjunction with the PCR array screening results. A total of 16 lncRNAs were found to be significant in the cancer survival and progression of HNC patients. Altogether, we established a 31-lncRNA signature panel that predicts HNC prognosis.

Upon further detailed investigation, we found some notable lncRNAs in our panel, including XIST, TSIX, HOXA11-AS, WT1-AS, IPW, and MALAT1, with significant dysregulation in multiple HNC cell lines. A study by Yao et al. also identified HOXA11-AS and MALAT1 as potential biomarkers for HNC (34). The prognostic risk of MALAT1 has also been well established in various previous studies (32, 35). Interestingly, although studies have deemed MALAT1 as an oncogene across many cancer types (35), our results indicated that it was downregulated in HNC. TCGA data analysis also showed that high expression of MALAT1 resulted in higher overall survival, which correlates with our study. Thus, these common lncRNAs may have high potential for future HNC-specific studies. On the other hand, some lncRNAs from our panel are relatively novel lncRNAs, such as WT1-AS, where only a handful of studies have proposed its carcinogenic function in lung, cervical, and breast cancer (36–38). Knowledge regarding HOXA11-AS is also sparse, although recent studies have elaborated on its role in liver (39), lung (40), head-neck (34), and other cancers (41). Not much is known about lncRNA IPW and TSIX either, but some preliminary studies have pointed out potential interactions between TSIX and the more well-known lncRNA XIST in regards to X chromosome modulation (42). Although some early studies predicted an inverse correlation between these two molecules (43), newer studies began to disprove their correlation, focusing on their individual functions instead (44).

Numerous studies have linked XIST with multiple cancer types, such as colorectal (45), lung (46), and breast cancer (47). Various cancers such as thyroid (48) and osteoscaroma (49) have also shown XIST to act as an oncogene, which coincides with our findings in HNC. Many of these studies have also suggested that XIST could act as a prognostic marker, given its various cancerous functions. Thus, we selected the lncRNA XIST for *in vitro* studies due to its performance in our screening results, along with its novelty in HNC. Additionally, various annotations from GO and KEGG, such as ‘adherens junction’, ‘focal adhesion’, and ‘cell-cell adhesion’, all strongly allude to the metastatic functions as well, which correlates with our functional analysis. Our results showed that XIST played essential roles in regulating cell migration and invasion, as silencing by siRNA significantly inhibited these functions in multiple HNC cells. These carcinogenic roles were also confirmed in previous cancer studies, such as liver (50), ovarian (51), and esophageal cancer (52). A review by Zhou et al. also highlighted the potential of XIST as a prognostic marker (53). Therefore, our findings implied that because XIST affected HNC progression through these metastatic functions, it can be used as a prognostic marker. Taken together, XIST can be used as an HNC marker that can predict prognosis, and can potentially be used as therapeutic target.

In conclusion, we established a systematic profiling method to screen for prognostic lncRNA markers in HNC. Our results from the *in silico* and *in vitro* combination analysis suggests that the panel of 31-lncRNA signatures may contribute to HNC tumorigenesis, and can provide valuable prognostic data. Additionally, XIST demonstrated carcinogenic functions in HNC, implicating its ability as a prognostic marker. Overall, our findings greatly contribute to the knowledge of HNC and prognosis, and can potentially be expanded to applications in precision medicine.

## Data Availability Statement

The original contributions presented in the study are included in the article/**Supplementary Material**. Further inquiries can be directed to the corresponding authors.

## Author Contributions

S-JT was responsible for experimental analysis, acquisition of data, and the writing of the manuscript. A-JC and S-JT were responsible for the conceptualization and design. A-JC and JC were responsible for resources, project administration, supervision and funding acquisition. S-JT, A-JC, and G-RY were responsible for the review of the submitted manuscript. All authors contributed to the article and approved the submitted version.

## Funding

This research was supported by the Ministry of Science and Technology (Most-107-2314-B-182A-062-MY3), and Chang Gung Memorial Hospital – Linkou Medical Center (CMRPG3K1571).

## Conflict of Interest

The authors declare that the research was conducted in the absence of any commercial or financial relationships that could be construed as a potential conflict of interest.

## Publisher’s Note

All claims expressed in this article are solely those of the authors and do not necessarily represent those of their affiliated organizations, or those of the publisher, the editors and the reviewers. Any product that may be evaluated in this article, or claim that may be made by its manufacturer, is not guaranteed or endorsed by the publisher.
