# Supplementary figures and images for: Systematic Analysis and Identification of Dysregulated Panel lncRNAs Contributing to Poor Prognosis in Head-Neck Cancer
Source: Front Oncol. 2021 Oct 18;11:731752. doi: 10.3389/fonc.2021.731752 (PMC8558550; doi:10.3389/fonc.2021.731752)

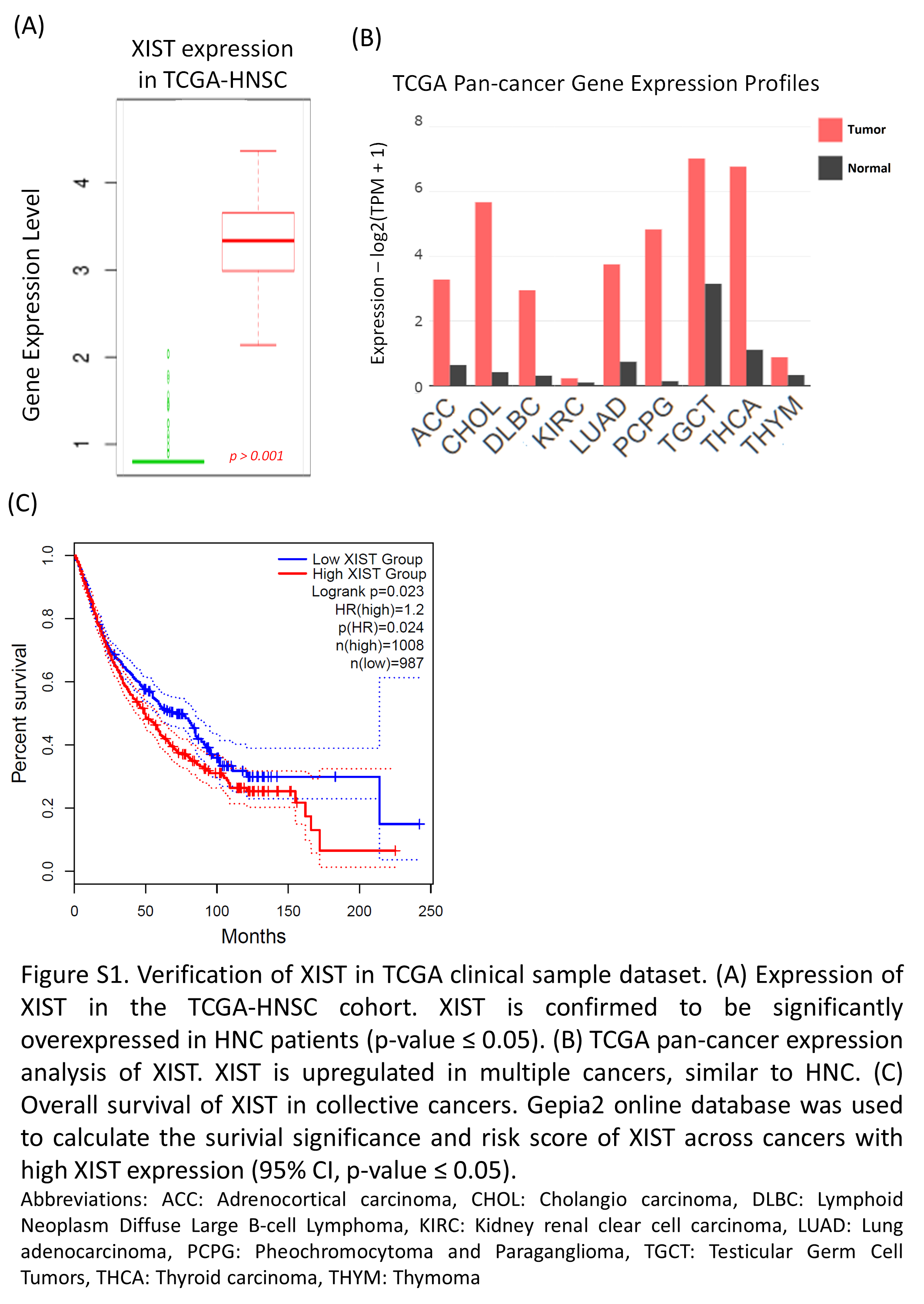

Supplement: Supplementary file 1 [file Image_1.tif]
